# Mouse Genome Database (MGD) 2019

**DOI:** 10.1093/nar/gky1056

**Published:** 2018-11-08

**Authors:** Carol J Bult, Judith A Blake, Cynthia L Smith, James A Kadin, Joel E Richardson, the Mouse Genome Database Group, A Anagnostopoulos, R Asabor, R M Baldarelli, J S Beal, S M Bello, O Blodgett, N E Butler, K R Christie, L E Corbani, J Creelman, M E Dolan, H J Drabkin, S L Giannatto, P Hale, D P Hill, M Law, A Mendoza, M McAndrews, D Miers, H Motenko, L Ni, H Onda, M Perry, J M Recla, B Richards-Smith, D Sitnikov, M Tomczuk, G Tonorio, L Wilming, Y Zhu

**Affiliations:** The Jackson Laboratory, 600 Main Street, Bar Harbor, ME 04609, USA

## Abstract

The Mouse Genome Database (MGD; http://www.informatics.jax.org) is the community model organism genetic and genome resource for the laboratory mouse. MGD is the authoritative source for biological reference data sets related to mouse genes, gene functions, phenotypes, and mouse models of human disease. MGD is the primary outlet for official gene, allele and mouse strain nomenclature based on the guidelines set by the International Committee on Standardized Nomenclature for Mice. In this report we describe significant enhancements to MGD, including two new graphical user interfaces: (i) the Multi Genome Viewer for exploring the genomes of multiple mouse strains and (ii) the Phenotype-Gene Expression matrix which was developed in collaboration with the Gene Expression Database (GXD) and allows researchers to compare gene expression and phenotype annotations for mouse genes. Other recent improvements include enhanced efficiency of our literature curation processes and the incorporation of Transcriptional Start Site (TSS) annotations from RIKEN’s FANTOM 5 initiative.

## INTRODUCTION

The Mouse Genome Database (MGD) is the community model organism knowledgebase for the laboratory mouse. MGD contains comprehensive information about mouse gene function, genotype-to-phenotype annotations, and mouse models of human disease (1). The mission of the MGD is to advance the use of the laboratory mouse as a model system for investigating the genetic and genomic basis of human health and disease. MGD maintains a comprehensive catalog of mouse genes and genome features connected to genomic sequence data and biological annotations. Annotations include (i) molecular function, biological process and cellular location of genes using terms and relations of the Gene Ontology (GO) (see Gene Ontology Consortium,^2^), (ii) mutations, variants and human disease models using terms from the Mammalian Phenotype Ontology (MP) and Disease Ontology (DO) and (iii) official nomenclature and identifiers for mouse gene names, symbols, alleles and strains (Table 1). The rigorous application of nomenclature and annotation standards in MGD ensures that the information in the resource is curated consistently to support robust and comprehensive data retrieval for sets of genes that share biological properties and data mining for knowledge discovery.

**Table 1.** Data for which MGD serves as an authoritative source

Data type	Description
Unified mouse genome feature catalog	MGD integrates predictions from Gencode and NCBI to generate a single, comprehensive catalog
Gene Ontology (GO) annotations for mouse	MGD expertly curates data from literature and integrates from others
Mouse Phenotype annotations	MGD expertly curates data from literature and integrates from large scale projects
Mouse models of human disease	MGD expertly curates mouse models of human disease using terms from the Disease Ontology
Gene to nucleotide sequence association	MGD collaborates with NCBI and Gencode
Gene to protein sequence association	MGD collaborates with UniProt and Protein Ontology
Mammalian Phenotype (MP) Ontology	MGD develops and distributes MP
Symbols, names, and stable accession identifiers for genes, alleles and mouse strains	MGD implements standards set by the International Committee on Standardized Genetic Nomenclature in Mice and coordinates with human and rat gene nomenclature committees

MGD is a core resource within the Mouse Genome Informatics (MGI) consortium (http://www.informatics.jax.org). Other database resources that are coordinated within the MGI consortium include the Gene Expression Database (GXD) (3), the Mouse Tumor Biology Database (MTB) (4), the Gene Ontology project (GO) (5), MouseMine (6), the International Mouse Strain Resource (IMSR) (7) and the CrePortal database of recombinase expressing mice (8). Data included in all resources hosted at the MGI website are obtained through a combination of expert curation of the biomedical literature and automated or semi-automatic processing of data sets downloaded from more than fifty other data resources. A summary of the current content of MGD is summarized in Table 2.

**Table 2.** Summary of MGD content September 2017–2018

Data type	2017	2018
Genes and genome features with nucleotide sequence data	47 693	49 244
Genes with protein sequence data	24 317	24 408
Mouse genes with human orthologs	17 089	17 094
Mouse genes with rat orthologs	18 509	18 512
Genes with GO annotations	24 502	24 581
Total number of GO annotations	312 109	316 240
Mutant alleles in mice	51 378	56 254
Genes with mutant alleles in mice	12 401	13 455
QTL records	6257	6605
Genotypes with phenotype annotation (MP)	60 951	62 551
Total number of MP annotations	315 657	326 292
Mouse models (genotypes) associated with human diseases	6027	6374
References in the MGD bibliography	237 578	258 926

In this report we describe significant enhancements to MGD, including two new graphical user interfaces: (i) the Multiple Genome Viewer for exploring the genomes of multiple mouse strains and (ii) the Phenotype/Gene Expression matrix which allows users to compare gene expression and phenotype annotations for mouse genes. Other improvements include improvements to literature curation processes, and the incorporation of TSS annotations from RIKEN’s FANTOM 5 initiative (9).

## NEW FEATURES AND CURATION WORKFLOW ENHANCEMENTS

### Multiple genome viewer

**Figure 1.**
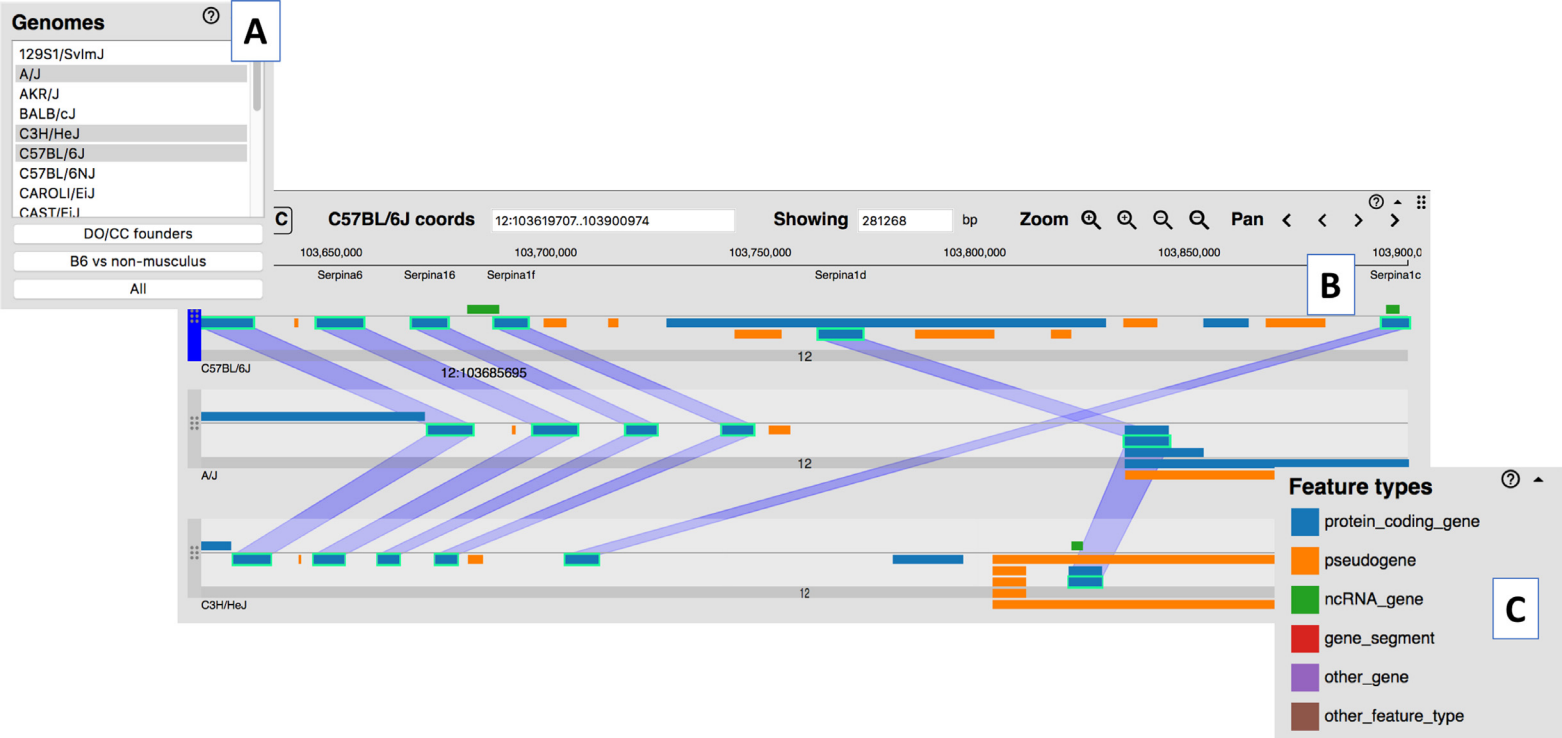
A screenshot of MGD’s Multiple Genome Viewer showing the display of genome annotations across multiple strains of mice. (**A**) Users may select one or more genomes to be displayed. (**B**) Equivalent genome features across the strains are highlighted by ‘swim lanes’ when a user clicks on one or more genome features. (**C**) Genome feature types (protein coding gene, pseudogene, etc.) are indicated by color; classes of genome features can be toggled on and off in the display.

The recent release of assembled and annotated genomes for 16 inbred mouse strains (https://www.biorxiv.org/content/early/2018/02/12/235838) and two wild-derived strains (CAROLI/EiJ and PAHARI/EiJ) (10) represent major milestones in mouse genetics and comparative genomics. MGD’s Multiple Genome Viewer (MGV; http://www.informatics.jax.org/mgv) was developed specifically to enable researchers to explore and compare chromosomal regions and synteny blocks between the C57BL/6J reference genome and the 18 other available mouse genomes (Figure 1). MGV shows corresponding regions of the user-selected genomes as horizontal stripes and the equivalent features in each genome via vertical connectors (Figure 1). The navigation of the genomes is synchronized as a user scrolls in 5′ or 3′ directions. Researchers can generate custom sets of genes and other genome features to be displayed in MGV by entering genome coordinates, function, phenotype, disease and/or pathway terms.

The genome feature annotations for the C57BL/6J genome displayed in MGV are taken from MGI’s Unified Mouse Genome Feature Catalog that integrates the genome feature annotations from Gencode, NCBI and miRBase into a single, non-redundant set (11). Currently, only the C57BL/6J assembly and annotations are ‘reference quality’, thus there are some gaps in the annotations which limit the ability of a user to identify equivalent genome features across all of the available genomes. As additional sequence data are generated, improvements will be made to the quality of all the assemblies and their corresponding genome feature predictions.

Gene model structure details and sequences for all 19 annotated mouse genomes are also accessible from MGI’s MouseMine (http://www.mousemine.org) through its user interface and web services (MouseMine web services back the Multiple Genome Viewer). Using MouseMine, researchers may search for genes in specific strains and retrieve relevant data including transcripts, exons in a GFF file, and CDSs in FASTA format. On a MouseMine gene page—or when viewing a list of genes—several new query templates are automatically run and provide easy navigation and retrieval of the structural components of gene models (e.g. exons, introns) across user-selected strains. These new templates provide access from a gene to its strain-specific genomic sequences, transcripts, CDSs, or exons. An Export button, located above a report, will allow a user to download results in several formats: tab or comma-separated file, FASTA or GFF3.

### Phenotype/Gene expression comparison matrix

**Figure 2.**
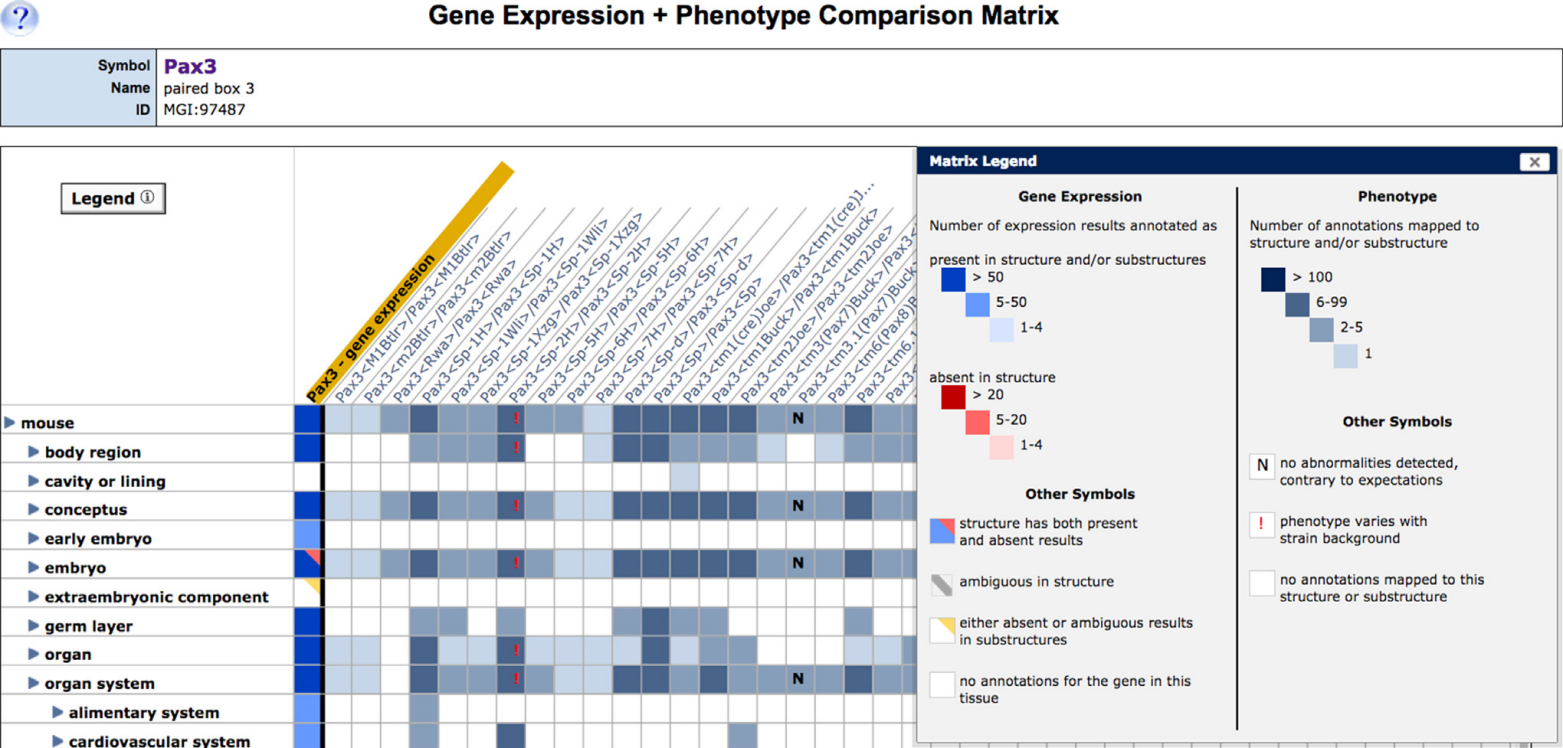
Screenshot of the Gene Expression-Phenotype Matrix. The first column (gold highlight) summarizes the wild-type expression pattern of the Pax3 gene. The color of matrix cells in the column indicates the type and number of expression annotations for each tissue; the conventions are defined in the matrix legend (inset). Genotype summary data associated with alleles of Pax3 are displayed in the adjacent columns. The tissues where each mutation/genotype has phenotypic effects are indicated by the presence of colored matrix cells. Cells with an ‘N’ indicate that an expected abnormality was not detected. A red exclamation point indicates the phenotype is affected by changes in mouse strain background. Clicking on blue toggles next to term names expands and collapses the anatomy vocabulary tree. Annotation details are displayed when users click in the cells of the matrix.

In collaboration with the Gene Expression Database (GXD), we deployed a new interface that allow users to compare gene expression and phenotype data for a given gene (see also Smith CM *et al.*, 12). The new Phenotype/Gene Expression Comparison Matrix, accessible from the Expression and Mutations, Alleles and Phenotype section of MGD’s gene detail pages, visually juxtaposes information about tissues where a gene is normally expressed against tissues where mutations in that gene cause abnormal phenotypes (Figure 2). Using this new data display tool researchers may explore the molecular mechanisms of disease by answering such questions as ‘What tissues affected by a gene mutation also show expression of that gene?’ or ‘What tissues affected by a gene mutation do not express that gene?’.

### Literature triage process improvements

While the numbers of publications indexed in PubMed that mention mice continues to grow (∼72 000 papers added in 2017), the subset of papers relevant to MGD (i.e. those focused on genetics and genomics of the laboratory mouse) has remained relatively stable. We curate ∼12 000 of these papers each year, primarily from a core set of 160 journals. A major challenge for MGD curators is how to identify the relevant subset of papers from a large corpus of biomedical literature. Manuscripts, while peer-reviewed, are often published without annotations using relevant bio-ontologies; authors often do not adhere to existing gene, allele or mouse strain nomenclature standards for example. As a consequence, the identification of publications that are actually relevant to MGD’s mission requires a substantial investment of time for manual review.

To improve the scalability of our literature curation efforts, we have streamlined our literature selection processes and implemented software infrastructure to support automation for these processes. We now store the full text of papers extracted from PDFs downloaded from publishers and assess the relevance of papers using keyword searches. Downloading papers from PLoS journals is performed automatically and takes advantage of the PLoS API’s full text search capabilities. Full text searches improve the identification of relevant papers for MGD because important keywords such as mouse and murine are often not mentioned in article titles and abstracts. In the eleven months following the implementation of the improved literature selection processes (aka, literature triage), individual curator efficiency in identifying papers relevant to mouse phenotypic alleles has increased by 83% as measured by number of relevant papers identified by an individual per unit time. For our user communities, the increased efficiency in literature curation means a shorter time between publication and accessibility of the phenotype and disease annotations from MGD.

To build training sets that can be used for future automatic efforts and to support research in natural language processing and machine learning, we also now store full text of papers that are deemed not relevant to MGD in addition to the relevant papers.

### Transcriptional Start Site (TSS) genome features

As part of MGD’s efforts to represent experimentally supported regulatory regions in the mouse genome, over 164 000 Transcriptional Start Sites (TSS) identified by investigators at the RIKEN Institute using Cap Analysis Gene Expression (CAGE) sequencing (9) were loaded into MGD. TSS sites are particularly informative for delineating the structure of promoter regions of genes; many genes have more than one promoter region that controls the expression of alternative transcript forms. Over 22 000 of the TSS sites identified by the RIKEN data are associated with annotated mouse genes. From a gene detail page, users can see the annotated TSS sorted by distance from the gene's 5′ end.

The RIKEN TSS data are also available as 1015 tracks in MGD’s JBrowse-based mouse genome sequence browser (http://jbrowse.informatics.jax.org/). The tracks implemented in JBrowse include TSSs obtained from sequencing primary cells (140 tracks), tissues (23 tracks), developmental stages (257 tracks), and time course experiments (591 tracks).

## IMPLEMENTATION AND PUBLIC ACCESS

The production database for MGD is a highly normalized relational database hosted on a PostgreSQL server behind a firewall. The production database is designed and optimized for data integration and incremental updating and is not directly accessible by the public. The public web interface is backed by a combination of a highly denormalized databases (also in PostgreSQL) and Solr/Lucene indexes, designed for high performance query and display in a read-only environment. The front-end data stores are refreshed from the production database once a week. The separation of public and production architectures provides a large measure of flexibility in project planning, as either side can (and often does) change without affecting the other.

MGD broadcasts data in a variety of ways to support basic research communities, clinical researchers and advanced users interested in programmatic or bulk access. MGD provides free public web access to data from http://www.informatics.jax.org. The web interface provides a simple ‘Quick Search’, available from all web pages in the system and is the most used entry point for users. The Quick Search may be used to search for genes and genome features, alleles and ontology or vocabulary terms. Multi-parameter query forms for a number of data types are provided to support searches based on specific user-driven constraints, Genes and Markers; Phenotypes, Alleles and Diseases; SNPs; and References. Data may be retrieved from most results pages by downloading text or Excel files, or forwarding results to Batch Query or MouseMine analysis tools (see below).

MGD offers batch querying interfaces for data retrieval for users wishing to retrieve data in bulk. The Batch Query tool (http://www.informatics.jax.org/batch) (13) is used for retrieving bulk data about lists of genome features. Feature identifiers can be typed in or uploaded from a file. Gene IDs from MGI, NCBI GENE, Ensembl, UniProt and other resources can be used. Users can choose the information set they wish to retrieve, such as genome location GO annotations, list of mutant alleles, MP annotations, RefSNP IDs and Disease Ontology (DO) terms. Results are returned as a web display or in tab delimited text or Excel format. Results may also be forwarded to MouseMine (see below).

MGD data access is available through MouseMine (http://www.mousemine.org), an instance of InterMine that offers flexible querying, templates, iterative querying of results and linking to other model organism InterMine instances. MouseMine access is also available via a RESTful API, with client libraries in Perl, Python, Ruby, Java and JavaScript. MouseMine contains many data sets from MGD, including genes and genome features, alleles, strains and annotations to GO, MP and DO.

MGD provides a large set of regularly updated database reports from http://www.informatics.jax.org/downloads/. Direct SQL access to a read-only copy of the database is also offered. Those interested in SQL access should contact MGI user support for an account. MGI User Support is also available to assist users in generating customized reports on request.

Interactive graphical interfaces for browsing mouse genome annotations is supported through our instance of JBrowse (http://jbrowse.informatics.jax.org/), a JavaScript-based interactive genome browser with multiple features for navigation and track selection (14).

MGD is one of the founding members of the Alliance of Genome Resources, a new data resource integration effort among the major model organism (MOD) database groups and the Gene Ontology Consortium (GOC). The other founding members of the Alliance are FlyBase, WormBase, Saccharomyces Genome Database (SGD), Rat Genome Database (RGD) and the Zebrafish Information Network (ZFIN). The Alliance is standardizing access to common data types from different model organisms to better support comparative biology investigations for biomedical researchers (15). Genetic and genomic data for the laboratory mouse that are curated by MGD are available from the public web portal for the Alliance (http://www.alliancegenome.org). Data types accessible from the Alliance web site currently include gene names and symbols, genome locations, orthology, function annotations, and disease associations. New data types (e.g. gene expression, interactions, etc.) are being added to the site regularly. The Alliance serves as one of the designated Data Stewards for the NIH Data Commons Pilot Project, providing access to model organism data and annotations via APIs to promote the development of the next generation of cloud-based data access and analysis platforms in genome biology.

## FUTURE DIRECTIONS

In addition to continuing the essential core functions of MGD, three major enhancements are planned for this resource over the next year. First, following the decision of NCBI’s dbSNP to no longer include variation data from model organisms, MGD will implement data loads for mouse SNP data from the European Variation Archive (EVA; https://www.ebi.ac.uk/eva/). Second, we will implement new user interfaces focused on delivering diverse data about individual mouse strains. Although we have provided a strain accession ID service and descriptions of strain characteristics from the classic Festing's inbred strain lists resource for many years (http://www.informatics.jax.org/inbred_strains/mouse/STRAINS.shtml), the new strain detail pages will provide access to detailed information about strain-specific mutations, phenotype and disease model information, published references, and links to multiple external resources such as Mouse Phenome Database (MPD) (16), the International Mouse Strain Resource (IMSR) (7) and MGD’s new Multiple Genome Viewer. Third, the Multiple Genome Viewer will be extended to support display of the intron/exon structure of protein coding genes to allow the comparison of gene structure across strains.

## OUTREACH

User Support staff are available for on-site help and training on the use of MGD and other MGI data resources. MGD provides off-site workshop/tutorial programs (roadshows) that include lectures, demos and hands-on tutorials and can be customized to the research interests of the audience. To inquire about hosting an MGD roadshow, email mgi-help@jax.org. On-line training materials for MGD and other MGI data resources are available as FAQs and on-demand help documents.

Members of the User Support team can be contacted via email, web requests, phone or fax.

- World wide web: http://www.informatics.jax.org/mgihome/support/mgi_inbox.shtml
- Facebook: https://www.facebook.com/mgi.informatics
- Twitter: https://twitter.com/mgi mouse and https://twitter.com/hmdc_mgi
- Email access: mgi-help@jax.org
- Telephone access: +1 207 288 6445
- Fax access: +1 207 288 6830

MGI-LIST (http://www.informatics.jax.org/mgihome/lists/lists.shtml) is a forum for topics in mouse genetics and MGI news updates. It is a moderated and active email-based bulletin board for the scientific community supported by the MGD User Support group. MGI-LIST has over 1800 subscribers. A second list service, MGI-TECHNICAL-LIST, is a forum for technical information about accessing MGI data for software developers and bioinformaticians, for using the APIs and for making web links to MGI pages.

## CITING MGD

For a general citation of the MGI resource, researchers should cite this article. In addition, the following citation format is suggested when referring to datasets specific to the MGD component of MGI: mouse genome database (MGD), MGI, The Jackson Laboratory, Bar Harbor, Maine (URL: http://www.informatics.jax.org). Type in date (month, year) when you retrieved the data cited.

## MOUSE GENOME DATABASE GROUP

A. Anagnostopoulos, R. Asabor, R.M. Baldarelli, J.S. Beal, S.M. Bello, O. Blodgett, N.E. Butler, K.R. Christie, L.E. Corbani, J. Creelman, M.E.Dolan, H.J. Drabkin, S.L. Giannatto, P. Hale, D.P. Hill, M. Law, A. Mendoza, M. McAndrews, D. Miers, H. Motenko, L. Ni, H. Onda, M. Perry, J.M. Recla, B. Richards-Smith, D. Sitnikov, M. Tomczuk, G. Tonorio, L. Wilming and Y. Zhu.
